# Twelve Essentials of Science-based Policy

**Published:** 2005-09-15

**Authors:** Bernard C.K Choi

**Affiliations:** Centre for Chronic Disease Prevention and Control, Public Health Agency of Canada; Dr Choi is also affiliated with the Department of Epidemiology and Community Medicine, University of Ottawa, Ottawa, Ontario, and the Department of Public Health Sciences, University of Toronto, Toronto, Ontario

## Abstract

This article presents a systematic framework of 12 essentials, or basic elements, of science-based policy. The 12 essentials are grouped into three categories, or areas, as follows: 1) knowledge generation, which includes credible design, accurate data, sound analysis, and comprehensive synthesis; 2) knowledge exchange, which includes relevant content, appropriate translation, timely dissemination, and modulated release; and 3) knowledge uptake, which includes accessible information, readable message, motivated user, and rewarding outcome.

## Introduction

The relationship between science and policy is an important topic in evidence-based public health policy and practice (1). It seems logical to assume that as scientific research generates more quality findings, policymakers will make better decisions. However, numerous underlying obstacles exist (2).

A systematic framework can be used to describe the key components that link science to policy. The framework, which consists of three areas that are subdivided into 12 essentials (basic elements), reveals issues and solutions related to science-based decision making. In this article, *policy* is defined broadly to include not only legislation but also "prudence or wisdom in the management of affairs" and "a definite course or method of action selected from among alternatives in light of given conditions to guide and determine present and future decisions" (3). Therefore, the term *policymakers* may encompass public health practitioners, public health researchers, and even the general public, because members of the general public make health decisions for themselves and their families.

Science-based policy involves producing high-quality scientific evidence, building bridges between the producers and users of scientific evidence, and incorporating scientific evidence into health policy and practice (4). Accordingly, the three primary areas in science-based policy are knowledge generation, knowledge exchange, and knowledge uptake (Table 1). Within these three areas, the 12 essentials are categorized as follows: *knowledge generation* — 1) credible design, 2) accurate data, 3) sound analysis, and 4) comprehensive synthesis; *knowledge exchange* — 5) relevant content, 6) appropriate translation, 7) timely dissemination, and 8) modulated release; and *knowledge uptake* — 9) accessible information, 10) readable message, 11) motivated user, and 12) rewarding outcome (Table 1).

The names of the three areas described in this framework vary in other articles. For example, *knowledge generation* (5,6) has also been called *knowledge acquisition* (7) and *knowledge creation* (8); *knowledge exchange* (6,9-11) has been called *knowledge dissemination* (7,8,12), *knowledge transfer* (9,11), *knowledge brokering* (10), *knowledge translation* (13), and *knowledge access* (5); and *knowledge uptake* (6,9) has been referred to as *knowledge application* (7,8), *knowledge utilization* (8,12), and *knowledge use* (5). The meanings of the terms vary slightly. For example, the term *dissemination* implies a one-way transmission of knowledge, whereas the terms *transfer* and *exchange* imply a two-way transfer of information (14). The term *brokering* seems to be associated with a process, the objective of which is to exchange information (10).

## Knowledge Generation

### Credible design

Ideally, evidence for policy decisions should be generated from scientific research based on high-quality study designs. In general, the strength of data generated by various study designs results in a hierarchical pattern. Experimental studies such as clinical trials and field trials provide strong evidence; community trials and observational studies such as cohort studies and case-control studies provide moderate evidence; other observational studies such as historical cohort studies, cross-sectional studies, and ecological studies provide weak evidence; and case reports and news reports provide minimal evidence (15-18).

However, the scientific evidence hierarchy is often turned upside down when policy decisions are being made. News reports and case reports often play an important role in policy decisions, because decision makers, including those in the general public, often do not have the time, ability, or expertise to access and synthesize the evidence from high-quality studies. For example, in 1999, the newspaper *USA Today* published the following health-related headlines: "'Scars' May Be Cancer Predictor," "Persistent Heartburn Is a Cancer Warning Sign," "Two Drinks a Day Keep Stroke Away," "Study: High-Fiber Diets Don't Cut Colon Cancer," and "No Link Found Between Fat, Breast Cancer" (19). News headlines can be based on inconclusive evidence (e.g., "*may* be"), scare tactics (e.g., "warning sign"), disregard of details (e.g., the health risks of drinking, such as liver disease), and conflicting messages (e.g., reporting results that are different from numerous other studies).

Even when scientific evidence is produced from adequately designed studies, current knowledge generation can be hindered by a *false-positive research cycle* (Figure) (20). Consider the following scenario. Evidence relating cellular telephone use and brain tumors is still inconclusive, despite the multiple studies that have been done and the widespread attention given to the topic (21). Assume the null hypothesis is true — that cellular telephone use does not cause brain tumors. In addition, assume that as a result of chance or bias (and not a high-quality study), some researchers report finding an association between cellular telephone use and brain tumors (a false-positive study). Publication of the false-positive study creates concern, and the problem becomes a hot topic (hot topic bias) which results in more studies — perhaps even 100 — that are designed to investigate the potential problem (22). At the conventional significance level, or a level, of .05, five of the 100 studies are expected to have positive results (23); in other words, five of the studies in this example would be expected to find that cellular telephone use causes brain tumors. The researchers who obtain positive results are more likely to document their results and submit papers to a journal (positive results bias), and journal editors are more likely to publish studies with positive results (editor's bias) (20). In other words, the five positive studies (which are actually false-positive studies) are more likely to be published, and few of the other 95 studies that did not find an association between cellular telephone use and brain tumors will be published (publication bias). The five additional false-positive studies make the topic even more urgent in the research community, and the false-positive research cycle begins again as more studies are designed to assess the problem. Through this biased process, researchers can often "prove" something out of nothing.

**Figure:**
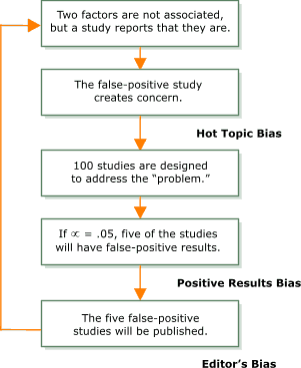
The false-positive research cycle..

### Accurate data

*Bias* is defined as the "deviation of results or inferences from the truth, or processes leading to such deviation" (24). The best way to identify bias is by comparing results with the truth, or a gold standard. For example, researchers conducted a study to determine the baseline accuracy of dentists' readings of dental radiographs (bitewings) (25). The study's methodology involved constructing 15 models of the posterior part of a natural dentition. The models had extracted teeth mounted in a medium with a radiodensity similar to that of human bone. Bitewing radiographs were taken of the simulated dentitions. The teeth used in the model mounts were removed from the models, serially sectioned, and examined with a microscope for caries. The results of the microscopic examination were established as the gold standard. Dentists were asked to read independently the 15 sets of bitewing radiographs and make treatment decisions about the teeth. The agreement between the dentists' readings and the gold standard established by the microscope results was poor (mean κ = 0.35) (25).

Even laboratory tests cannot guarantee the accuracy of a study's data. For example, many physicians use four different types of laboratory tests to diagnose leukemia (routine morphology testing, electron microscopy, cell surface marker identification, and cancer cytogenetics), and the four test results often seem contradictory. An interrater agreement study was conducted, with each of the four laboratories being classified as a rater. The interrater reliability result confirmed that the results were contradictory. Results from the four diagnostic laboratories correlated poorly for cell type identification in leukemia (pairwise κ = 0.17–0.40) (26).

Health data often come from health surveys using questionnaires, and the accuracy of the data is likely affected by questionnaire biases. For example, questions or answers may be phrased in a way that misleads respondents and causes them to make an incorrect choice (framing bias) (27). An example of framing bias follows:


*Which operation would you prefer?*
[ ] An operation that has a 5% mortality[ ] An operation that 90% of the patients will survive

People may choose the second option when they read "90%" and "survive," even though a 90% survival rate (which is a 10% mortality rate) is actually worse than a 5% mortality rate.

According to a comprehensive assessment of 109 instances of bias that were found in scientific research (literature review, 4; study design, 31; study execution, 3; data collection, 46; analysis, 15; interpretation, 7; publication, 3), most of the instances of bias were found in the data collection phase of research (46 of 109, or 42%, of the total instances) (22).

### Sound analysis

Failure to control for confounding effects is a common problem in data analysis. A *confounder* is a factor "that can cause or prevent the outcome of interest, is not an intermediate variable, and is associated with the factor under investigation" (24) is a factor "that can cause or prevent the outcome of interest, is not an intermediate variable, and is associated with the factor under investigation" (24). For example, if researchers were studying the association between drinking alcohol and lung disease, they would need to treat smoking as a potential confounder because 1) smoking is known to cause lung disease, and 2) smoking and drinking alcohol are often associated behaviors. Techniques to control for confounding include stratification and mathematical modeling (28,29).

Failing to conduct a sound data analysis could completely change the results of a study. In a mass screening for colorectal cancer, Zheng et al evaluated the accuracy of occult blood testing, using rectoscopy as the gold standard for comparison (30). Clinical and epidemiological data from 60,496 individuals were collected. It was found that of the 477 individuals who had colorectal cancer diagnosed by rectoscopy (the gold standard), 437 were identified as having colorectal cancer by the occult blood test. This corresponded to a test sensitivity of 92% (437/477), which indicated that the occult blood test was a good screening test for colorectal cancer. The results were submitted to a scientific journal, and comments from two reviewers were received. One reviewer was pleased with the study and recommended publication. The other reviewer pointed out a gross error in the calculations and mentioned "work-up bias." According to the original paper on work-up bias, it is not an easy issue to address (31). An appropriate mathematical procedure was subsequently developed to address the work-up bias (32). Using the new procedure, the occult blood test sensitivity was recalculated to be 28%, indicating that it was not a good screening test for colorectal cancer (30). Therefore, the proper analysis completely reversed the study's conclusion.

### Comprehensive synthesis

Scientific papers are being published constantly. Approximately 30,000 biomedical journals are being published currently, and 17,000 new biomedical books are published every year. On average, physicians would have to read 19 articles each day to stay knowledgeable about new developments in their field (33,34). Comprehensive syntheses of current information are needed to address potential problems such as lack of time and lack of expertise (35). Comprehensive syntheses include narrative reviews, systematic reviews, meta-analyses, meta-databases, inventories of best practices, and public health observatories. They make life easier for consumers of scientific material, such as scientists, physicians, and policymakers, collectively referred to as *knowledge users* in this article.

A narrative review is a summary of the literature that exists on a particular topic; informal and subjective methods are used to collect and interpret information (33,36). A systematic review is a summary that is written after a comprehensive search for relevant studies and then evaluated and synthesized according to a predetermined and an explicit method (33,37,38). A meta-analysis (an analysis of several analyses) takes a systematic review one step further by mathematically aggregating available data from independent studies to yield a more statistically powerful estimate (33,36,39). A meta-database (a database of several databases) includes information about the location, source, content, and other details of the relevant databases (40). An inventory of best practices (or better practices) is created using an approach based largely on less rigorous study designs of practices and programs. The inventory often focuses on particular organizational behaviors for which conclusive quantitative evaluations are difficult to design and execute (41). A public health observatory is more detached from actual health phenomena and events, provides objective descriptions and analyses, and provides forecasting of patterns, interrelationships, processes, and public health outcomes (42,43).

Comprehensive syntheses can be a major undertaking. For example, a lifestyle modification guide was created to prevent and control hypertension. It was a 50-page supplementary issue of a scientific journal based on a review of 37 years (1960 to 1996) of scientific literature on weight, alcohol, exercise, sodium, calcium, magnesium, potassium, and stress and their effects on the body (44).

Some comprehensive syntheses require a review of not only contemporary literature but also historical literature. For example, one analysis was composed of 12 lessons for public health surveillance in the twenty-first century. The lessons were created after conducting a broad review of the historical documents on major epidemics during the past 5000 years (since 3180 BC) and included the plague, smallpox, dancing mania, cholera, the Spanish flu, and lung cancer (45).

## Knowledge Exchange

### Relevant content

Information should not be disseminated all at once and should not be provided to everyone. Only relevant information needs to be disseminated. For example, depending on the audience, one of two information dissemination approaches can be used: the encyclopedia approach or the fire-alarm approach (46). The encyclopedia approach involves conveying all available information in the form of reports, atlases, Web sites, and other methods. This type of information is needed by knowledge users such as scientists and certain policymakers who need extremely detailed information.

For most policymakers and the general public, the fire-alarm approach may be more appropriate. This approach involves only conveying information when selected indicators are not in the normal range and indicate a potential problem. For example, it has been proposed that new composite indicators for public health, similar to economic indicators such as the Dow Jones average or the consumer price index, be developed to document the relevant health information needed for public health decisions (20,47). Many stockholders trade successfully by buying or selling their stock holdings based on the performance of economic composite indicators. In a similar way, indicators such as a national health index, national heart health index, and national diet index could be helpful to health policymakers.

### Appropriate translation

As scientists make new discoveries, more sophisticated methods and theories are developed. At some point, the average policymaker and even some scientists cannot understand the information. The key is to strike a balance between providing all available information and providing what is needed by knowledge users.  "Complex models with simple model-user interface" can be used to achieve this goal (48). Following is an example of how such a model-user interface was created for public health practitioners.

During the first months of the severe acute respiratory syndrome (SARS) epidemic in 2003, a mathematical model was developed to predict the spread of SARS (49). The mathematical model can be thought of as a machine, with the engine of the machine comprising a series of four mathematical equations:

*C_ti_* = *R_0_^t^*

*C* = Σ *C_ti_*

*D_ti + d_* = *C_ti_* × *F*

*D* = Σ *D_ti + d_*

where *C*_*ti*_indicates the predicted number of incident cases on day *ti,* and *t* is time expressed in the number of incubation periods; *C,* the predicted total number of cases; *D_ti + d_,* the predicted number of deaths on day *ti + d*, and *t* is time expressed in the number of incubation periods; *D,* the predicted total number of deaths; *R_0_,* the basic reproductive number (i.e., the expected number of new infectious cases per infectious case); *F,* the case-fatality rate (i.e., the proportion of cases who die within the symptomatic period); *i,* the incubation period (i.e., the time from infection to symptoms); and *d,* duration of disease (i.e., the time from symptoms to recovery or death).

These equations are complex but do not have to be understood to be used, just as a person who drives a car does not have to understand how the engine works. The model-user interface is simple. The required information for using the previous SARS model to predict the number of SARS cases and deaths consists of only *R_0,_ F, i, *and *d, *and the result is a set of several line graphs showing the predicted and observed numbers of SARS cases and deaths. The deviation of the observed numbers from the predicted numbers indicates the success of infection control measures (49).

For the general public, an effective yet simple and basic way to convey, or translate, complex information is by using health proverbs (50). Sayings such as "an apple a day keeps the doctor away" (51) have helped convey important health messages through the years. They were created by our ancestors, and we have the responsibility to create new science-based health proverbs for future generations.

Public health practitioners can learn about knowledge translation techniques from weather forecasters (52), who use symbols (such as a sun partly covered by clouds) and maps to explain the weather. Symbols could be used to denote public health events, and the public could receive short- and long-term public health forecasts and public health alerts, complete with color-coded maps to illustrate public health problems in space and time.

### Timely dissemination

Timely dissemination of information requires an ongoing information distribution mechanism. For example, 365 health indicators relevant to the general public could be developed, with one per day being discussed on the evening news (20). After the news and the weather forecasts, the reporter could discuss one of the indicators, such as air pollution during the previous 5 years and its predicted relationship to asthma in the next 3 years. The public would not be expected to watch the news without fail, but if the information dissemination occurred daily, the public's awareness and knowledge would increase with time (53).

In Canada, approximately 167,456 deaths result from chronic diseases each year. A *chronic disease clock* was developed by the Public Health Agency of Canada to disseminate information in real time on its Web site (54). The chronic disease clock is a digital clock with two categories: *chronic-disease–related deaths so far this year* and *chronic-disease–related deaths so far today (as of 12:00 midnight)*. People can actually watch the number of deaths attributable to chronic disease increase every few minutes because one death occurs every 3 minutes in Canada. The clock keeps running 24 hours per day, 365 days per year.

### Modulated release

The general public is overwhelmed by health information. The end result is that they do nothing to improve their health because they do not know how to begin the process. The various types of available information need to be prioritized and disseminated in stages.

For example, the World Health Organization's *The World Health Report* contains an immense amount of information (55). Chapter 4 of *The World Health Report 2002* is about major health risks. In industrialized countries, the leading risk factors for chronic diseases are tobacco use, high blood pressure, excessive alcohol consumption, high cholesterol, overweight, low fruit and vegetable intake, and physical inactivity. The four major chronic diseases in terms of resulting disability are cardiovascular disease, cancer, chronic respiratory diseases, and neuropsychiatric disorders (55). The information in the chapter can be prioritized for modulated release (56) in three steps. To promote health, the public is told to *play it SAFE* (with the acronym SAFE representing smoking, alcohol, food, and exercise) — refrain from smoking, drink alcohol in moderation, eat a balanced diet, and increase physical activity. If they do not play it SAFE, they have to *call a COP* to assess the situation (with COP representing cholesterol, obesity, and pressure) — go for annual medical examinations to assess blood cholesterol levels, weight, and blood pressure. If they do not play it SAFE and call a COP, they have to *expect HARM* (with HARM representing heart disease, abnormal growth, respiratory disease, and mental disorders) — in the form of chronic conditions such as heart disease, cancer, lung disease, and mental disorders. They would then have to seek treatment. SAFE–COP–HARM concisely summarizes the important information (56).

## Knowledge Uptake

### Accessible information

Scientific findings must be published in accessible formats. For example, information posted on a Web site may be considered accessible; however, some people do not have access to the Internet. Even people who do have Internet access may have difficulty retrieving a specific piece of information. For example, a Google search of the Internet using the key words *health* *information* resulted in 13,200,000 Web sites (53).

Various unique information dissemination tools have been invented. For example, executives at Xerox's Palo Alto Research Center (Palo Alto, Calif) can monitor the company's overall share price by watching an office fountain. The water flow is controlled through an Ethernet connection to a computer that has the latest stock data. Flow strengthens when the price increases (57).

New ways to actively market information and make it accessible to various populations are needed. A group of experts at an occupational health workshop for Latin Americans suggested unique ideas such as writing folk songs for the radio on the health effects of pesticides and organizing concerts with themes related to healthy living (58). The Brazilian Ministry of Health distributes a free package of two decks of playing cards, and one health message is written on each card, for a total of 104 health messages. Messages include tips such as "Take a walk with your dog for 30 minutes to burn up to 200 calories" and "Increase your fruit and vegetable consumption to five times a day." Other ways to make information accessible include incorporating messages into theatrical performances and story-telling sessions (53).

### Readable message

To be understood by different audiences, a message must be conveyed in relevant terms. For example, Canadian policymakers readily understand the economic and health impact of smoking on society. For them, a relevant message would be that eliminating tobacco use for 1 year in Canada would save $16.5 billion and prevent 47,000 deaths per year (59). This message may not be relevant to members of the general public who are not interested in policy and economics but are passionate about sports. Instead of telling them about how much society will save if they quit smoking, you could tell them how many important sports events, such as Stanley Cup hockey playoffs, World Cup international soccer games, or Super Bowl football playoffs, they would miss in their lifetime if they continued smoking (59).

For younger audiences, a relevant message such as "smoking makes you ugly" could be an innovative way to convey smoking-related information (60). Teenage smokers who do not care about the long-term health effects of tobacco smoking may be able relate to the more immediate effects on appearance, such as smoking-induced facial wrinkles (61,62) and baldness (63).

### Motivated user

It is important to raise awareness of how scientific evidence can be used to make health policy decisions. The key is to create an atmosphere in which knowledge users are interested in and seeking out scientific knowledge rather than being inundated with unwanted information. Knowledge users can be motivated in many ways, and education plays an important role. Presenting facts is not enough. For example, after returning home from a doctor's office, a colleague's teenage son told her that his doctor told him he was obese. The boy then said that he really did not need to worry about the problem because obesity was so common. The boy had the facts but was not motivated to do anything about them.

Educating people by teaching them about the severity and consequences of a health problem helps motivate them to act. For example, obese people need to know that they have a higher risk of developing chronic diseases such as diabetes and heart disease. Knowing the number of people who became blind or had limbs amputated because of diabetes would be a better way to drive home the ramifications of diabetes than simply stating the number of people who had diabetes.

### Rewarding outcome

Policymakers and the general public must be convinced that using science for making health decisions will be beneficial and have a noticeable impact on their health — in other words, that it will have a rewarding outcome. For example, mathematical prediction models could help policymakers evaluate how various policies will affect a particular situation. To help show the general public how scientific evidence can be used to make health decisions and improve their health, computer software could be used to calculate the probability of disease risks or overall health outcomes based on input related to personal lifestyle choices, demographics, diet, and smoking (20). For example, a 20-year-old man in excellent health may find out that he is expected to live 75 years. The computer program could be used to show him that if he were to start smoking, he would only be expected to live 67 years (64). The 8-year difference may be rewarding enough for him to decide not to start smoking.

## Conclusion

The science-based policy framework of knowledge generation, knowledge exchange, and knowledge uptake has similarities to Boyer's research (65). Boyer studied the concept of scholarship and distinguished four kinds of scholarly pursuits: discovery, integration, application, and teaching (65). Many parallels exist between Boyer's work and the framework described in this article: Boyer's discovery category parallels the framework's knowledge generation area, his integration category parallels the knowledge exchange area, and his application category parallels the knowledge uptake area. Overall, education is important in all three areas of the framework.

Corresponding to the 12 essentials are 12 recommendations for the future (Table 2). It is hoped that these recommendations will stimulate additional research and provide evidence for the necessity of a strong evidence base in public health policy.

## Figures and Tables

**Table 1:** Three Areas and Twelve Essentials of Science-based Policy

**Knowledge Generation **	**Knowledge Exchange **	**Knowledge Uptake **
1. Credible design 2. Accurate data 3. Sound analysis 4. Comprehensive synthesis	5. Relevant content 6. Appropriate translation 7. Timely dissemination 8. Modulated release	9. Accessible information 10. Readable message 11. Motivated user 12. Rewarding outcome

**Table 2:** Twelve Recommendations for the Future of Science-based Policy

**Area**	**Essential**	**Recommendation**
Knowledge generation	Credible design	Use high-quality study designs and apply a systematic approach in research to prevent the false-positive research cycle.
	Accurate data	Apply existing methods and develop new methods for reducing bias and increasing data accuracy obtained from scientific research.
	Sound analysis	Apply sound analysis methods to produce high-quality results from scientific research.
	Comprehensive synthesis	Use existing tools and develop new tools for summarizing scientific findings.
Knowledge exchange	Relevant content	Apply existing methods and develop new methods to extract relevant content from existing information.
	Appropriate translation	Develop new techniques for information translation, and simplify the science–user interface.
	Timely dissemination	Develop innovative ways to disseminate information in a timely way.
	Modulated release	Create new methods for organizing the release of prioritized information.
Knowledge uptake	Accessible information	Invent new ways to market health information and make it more accessible.
	Readable message	Produce information in a readable, understandable format that is relevant to the audience.
	Motivated user	Educate and motivate policymakers so that they actively seek out scientific evidence to make decisions.
	Rewarding outcome	Develop new ways to effectively show how using science to make decisions is beneficial.
